# Investigating the Effects of Stove Emissions on Ocular and Cancer Cells

**DOI:** 10.1038/s41598-019-38803-4

**Published:** 2019-02-12

**Authors:** Bedia Begüm Karakoçak, Sameer Patel, Nathan Ravi, Pratim Biswas

**Affiliations:** 1 Department of Energy, Environmental, and Chemical Engineering, Washington University in St. Louis, St. Louis, MO 63130 USA; 2 Department of Ophthalmology and Visual Sciences, Washington University in St. Louis, St. Louis, MO 63110 USA; 3 Veterans Affairs St. Louis Hospital, St. Louis, MO 63106 USA

## Abstract

More than a third of the world’s population relies on solid fuels for cooking and heating, with major health consequences. Although solid fuel combustion emissions are known to increase the prevalence of illnesses such as chronic obstructive pulmonary disease and lung cancer, however, their effect on the eyes is underexplored. This study assesses the acute toxicity of solid fuel combustion emissions on healthy ocular cells and a cancer cell line. Three healthy ocular cell lines (corneal, lens, and retinal epithelial cells) and a cancer cell line (Chinese hamster ovary cells) were exposed to liquid and gas phase emissions from applewood and coal combustion. Following the exposure, real-time cell attachment behavior was monitored for at least 120 hours with electrical cell impedance spectroscopy. The viability of the cells, amount of apoptotic cells, and generation of reactive oxygen species (ROS) were quantified with MTT, ApoTox-Glo, and ROS-Glo H_2_O_2_ assays, respectively. The results showed that coal emissions compromised the viability of ocular cells more than applewood emissions. Interestingly, the cancer cells, although their viability was not compromised, generated 1.7 to 2.7 times more ROS than healthy cells. This acute exposure study provides compelling proof that biomass combustion emissions compromise the viability of ocular cells and increase ROS generation. The increased ROS generation was fatal for ocular cells, but it promoted the growth of cancer cells.

## Introduction

Nearly three billion people still use solid fuels, such as biomass, coal, and cow dung cakes, in inefficient stoves for cooking and heating. These stoves generate pollutants such as particulate matter (PM), CO, and CH_4_. The resultant exposure to household air pollution (HAP) has been associated with respiratory^1^ and cardiovascular diseases^2^, as well as formation of cataracts^3,4^. The World Health Organization (WHO) recognizes HAP as the single most significant health risk, accounting for 4.3 million premature deaths in 2012^5^.

Epidemiological studies have investigated associations between stove emissions and different health indicators, such as cardiovascular risk, hypertension, and lung function^3,4,6–8^. However, epidemiological associations between indoor air pollutants and morbidity and mortality are often hindered by relatively small sample sizes, which are frequently not considered representative, and by the logistical difficulties of fieldwork in developing countries. A truly fundamental approach to understand and characterize the human health burden related to indoor stove exposure would be to investigate effects both *in-vitro* and *in-vivo*.

**Figure 1:**
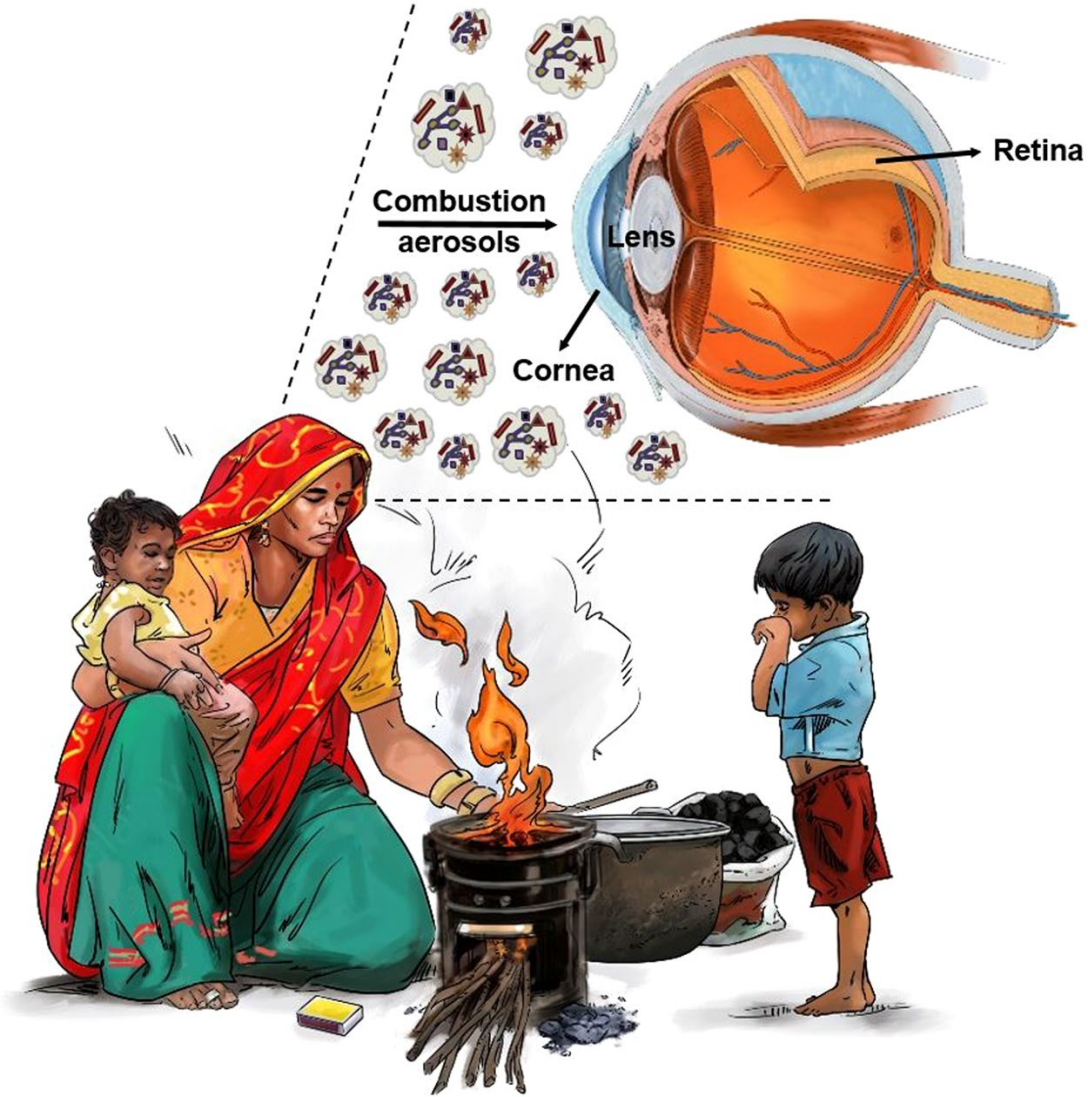
Daily activities like cooking and heating pose a threat, especially to women and children whose eyes are in direct contact with combustion smoke.

Both *in-vitro* and *in-vivo* studies centered on the respiratory system have shown that emissions from biomass fuels have carcinogenic and mutagenic properties^9–14^. However, on the cellular level, no study has investigated the possible adverse effects of exposure to solid fuel combustion emissions on the eye, a part of the central nervous system (Fig. 1). Only a few *in-vivo* studies have explored the effects of combustion smoke on the eye^15,16^. One *in-vivo* study showed that combustion smoke inhalation injury is caused by hypoxia and particulate matter acting alone or in combination^16^. How inhaling stove emissions causes secondary injury to the eye has not been fully explored; however, there is evidence that in response to smoke exposure from cotton burning, the permeability of the ocular blood vessels increased, which resulted in edema in the retina^16^.

Exposure to stove emissions can induce oxidative stress because it depletes the antioxidant protection against cataract formation^4^. On the cellular level, oxidative stress can lead to the subsequent release of reactive oxygen species (ROS), which damages both nuclear DNA and mitochondrial DNA^17–19^. Thus, biochemical and molecular changes occur which may lead to apoptosis (cell death) or tumor initiation^20^. The ROS handling capacities of healthy cells and cancer cells are different, and cancer cells are known to adapt well to oxidative stress^21^. On the other hand, healthy ocular cells handle oxidative stress differently. For example, unlike corneal and lens epithelial cells, retinal cells can cope well with oxidative stress due to their unique ROS handling capacity, an evolutionary mechanism for tolerating light exposure^16^. In view of this information, and because biomass and coal combustion is a repetitive daily activity for women, especially in developing countries^22–26^, there are clear motives to investigate the effect of biomass and coal combustion smoke at the cellular level first. The present study sought to determine whether ocular cells, as well as cancer cells, would be affected by exposure to emissions from applewood and coal combustion in a stove. To our knowledge, this is the first evaluation of the *in-vitro* toxicity of biomass and coal combustion emissions on the eye.

## Methods

**Figure 2:**
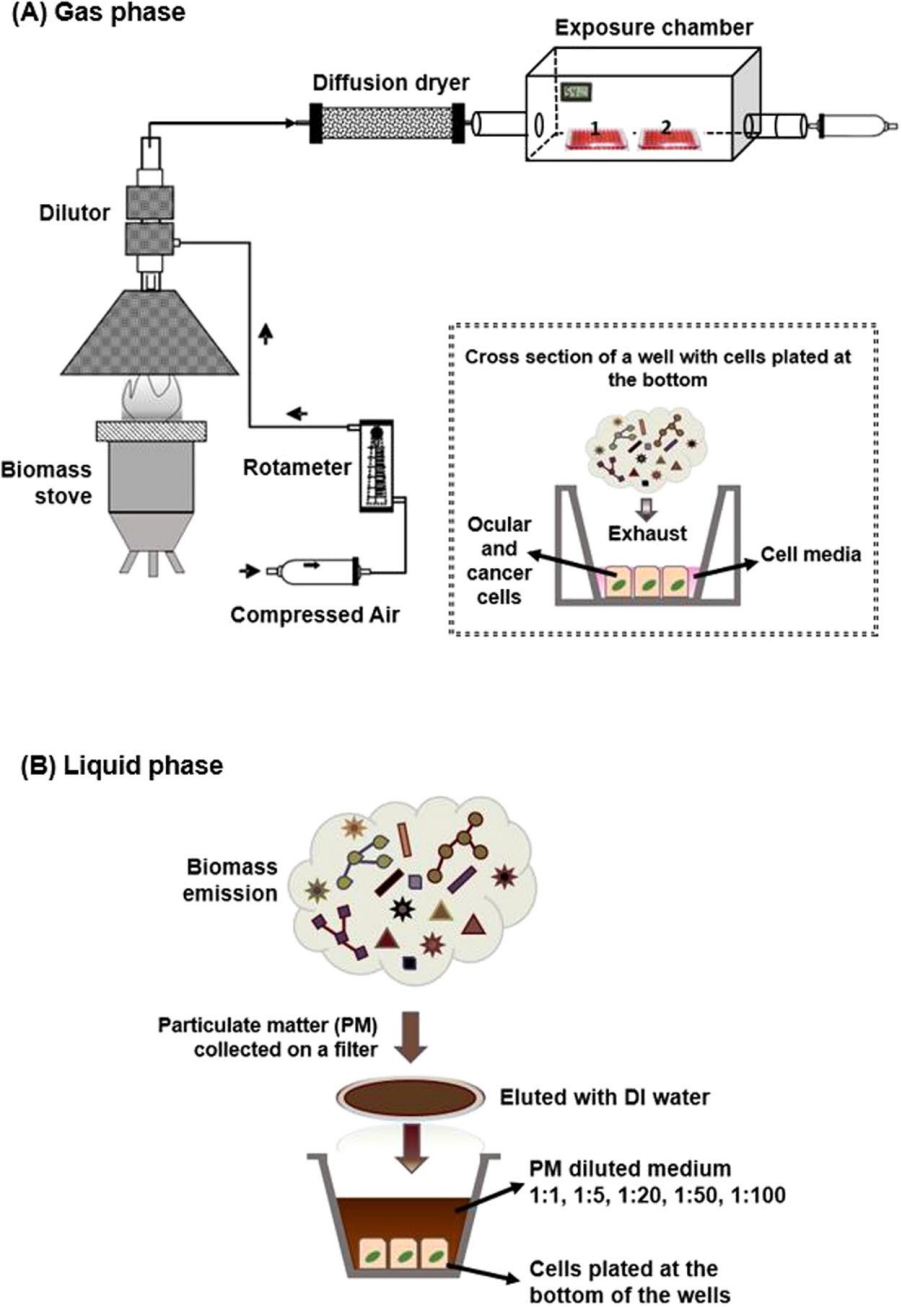
Schematic illustration of the experimental setup (**A**) Gas phase exposure. The inset represents the air-liquid interface created for the gas phase exposure. (**B**) Liquid phase exposure.

We conducted laboratory experiments to investigate the effects of stove emissions on ocular and cancer cells exposed via two pathways: (1) gas phase and (2) liquid phase. *In-vitro* models can be designed to closely mimic real exposure conditions; i.e., creating an air-liquid interface^27^. When used with a direct particle-to-cell deposition system, *in-vitro* models provide more physiologically relevant conditions for evaluating the cellular reactions, i.e., apoptosis initiation and ROS generation, in response to environmental pollutants^27–29^. In the first part of our study, to closely simulate ocular cell exposure to air pollutants, we performed gas phase exposure to cells maintained in “air-liquid interface” conditions, where only a thin layer of fluid separates the cells from the aerosols (Fig. 2A). Unlike the liquid phase exposure experiments, these conditions expose the target cells to both particulate and non-particulate constituents of the exhaust in naturally occurring proportions. In the second part of the study, liquid phase exposure experiments were conducted where particulate matter extracts were exposed to submerged cell cultures (Fig. 2B).

Coal and applewood were the two fuels used in this study. A laboratory hood setup (Fig. 2) with an aspiration-based dilution system was used to capture the emissions from fuel burned in a micro-gasifier stove. Comprehensive details about the stove, fuel properties, and their combustion and emissions characteristics were reported in previous studies^30,31^.

### Particulate matter collection and characterization

An improved gasifier cookstove, described in a previous study^30^, was used to burn applewood and coal. Detailed physical and chemical characteristics of the cookstove emissions from applewood and coal have also been reported in previous work^30^, and therefore are not repeated here. A forced-draft gasifier cookstove (Phillips, Model HD4012 LS) was used. Applewood chips with no additives were purchased locally (St. Louis, MO) and bituminous coal was procured from Brilliant, Alabama.

For gas phase exposure, cell culture plates were placed inside a chamber as illustrated in Fig. 2A. A minimum amount of medium was left in the wells to prevent the cells from drying out and to create an air-liquid interface for the exposure experiment (inset of Fig. 2A). During the exposure, the humidity of the chamber was maintained at 67% by placing wet towels inside the box (Table S1). Air, either alone (as the control) or containing cookstove emissions, was passed through the chamber for one hour at the same flow rate of 3 LPM as used for PM collection via a filter (Fig. 2). At the end of the exposure, the cell plates were removed from the chamber, and the cell culture medium was replenished.

For liquid phase exposure, the particulate matter (PM) emissions were first collected on a 47 mm Teflon filter (Sigma-Aldrich, St. Louis, MO) as illustrated in Fig. 2B. To extract the collected PM, the filter was submerged in 100 ml of deionized water and sonicated for 30 minutes. This extract was then further diluted to different strengths with Dulbecco’s modified Eagle’s medium (DMEM F-12) (Sigma-Aldrich, St. Louis, MO) with 10% fetal calf serum (Sigma-Aldrich, St. Louis, MO) and 1% antibiotic-antimitotic solution (Sigma-Aldrich, St. Louis, MO). Particle suspensions (1000-200-50-20-10 µg/ml, denoted in the rest of the study as dilution ratios of 1:1, 1:5, 1:20, 1:50, 1:100, respectively) were sonicated with a Branson sonicator bath (Hach, Loveland, CO) for 30 seconds immediately before being added to cell cultures. The cell media was removed prior to the applewood extract exposure in the liquid phase. The volume of applewood extracts was verified to be 150 μl in each well.

### Cell models

The selection criterion for cell lines was the likelihood of the cells being in direct environmental contact with the biomass emission. As illustrated in Fig. 1, biomass smoke is expected to hit the cornea first. Epidemiological studies have reported a direct correlation between cataract formation and biomass smoke exposure^3,4,32,33^, so lens epithelial cells were also included in this study. Further, retinal pigment epithelial cells have been reported to be adversely affected by cigarette smoke exposure^34–36^, so they were included in this study as the last healthy ocular cell line. On the other hand, the *in-vitro* toxicity of biomass smoke to lung cancer cell lines is well documented^10,37^; therefore, lung cancer cells were not assessed in this study. Instead, we chose an ovarian cancer cell line which has been shown to have a high mitotic index^38,39^ and also has not been studied before for *in-vitro* toxicity assessment of biomass smoke emissions.

Corneal epithelial cells (ATCC® PCS¬ 700-010^TM^), lens epithelial cells (ATCC® CRL¬ 11421^TM^), retinal pigment epithelial cells (ATCC® CRL¬ 2302™), and Chinese hamster ovary (CCL¬ 61™) cells were purchased from American Type Culture Collection (Manassas, VA).

### Cell culture conditions

In flat-bottom 96-well plates, 150 μl volumes of cells (2.0 × 10^4^ cells per well) were incubated at 37 °C in 5% CO_2_ until confluent (Figs S1 and S2), then exposed to biomass emissions. For the gas phase experiments, a minimum amount of cell medium was kept in each well to prevent cells from drying, while also ensuring direct contact with the biomass emission. For liquid phase experiments, biomass emission extracts were dispersed in cell culture medium (DMEM/F12) to be used for cell exposure, which was similarly kept with a thin layer of cell media to prevent the cells from drying.

### Assessment of cytotoxicity measurements with MTT, ApoTox-Glo, and ROS-Glo H_2_O_2_ assays

Cell metabolic activity, and hence the viability of cells in the presence of biomass smoke, was assessed with MTT (3-[4,5 dimethyl-thiazoly-2-yl] 2-5 diphenyl tetrazolium bromide).

The apoptotic cell amount and ROS generation, respectively, were measured by ApoTox-Glo and ROS Glo H_2_O_2_ assays. In all biological and imaging tests, cells exposed to filtered air alone served as a negative control. For the MTT assay, following the gas and liquid phase exposures, 100 µL of MTT (1 mg/l in growth media) was added to each well, and the plate was incubated for an additional 5 h at 37 °C in 5% CO_2_. The resulting blue component, produced by the reduction of the tetrazolium salt of MTT by mitochondrial dehydrogenase enzyme, was dissolved in 100 µL dimethyl sulfoxide (DMSO). The optical density of the colored product was read photometrically, using a spectrophotometer at 540 nm with a microplate reader (Molecular Devices Spectra Max 190). The absorbance of untreated cells was used as the negative control. The percentage viability of the cells was calculated from the ratio of the mean optical density of the sample to the optical density of the negative control^40^.

For the apoptosis and ROS detection experiments, the cells were cultured and exposed to liquid and gas phase biomass emissions in 96-well, clear-bottomed white plates. Caspase 3/7 activity, the key indicator of apoptosis, was evaluated using the ApoTox-Glo triplex assay according to the manufacturer’s protocol (Promega Biosciences San Luis Obispo, CA). The light output, measured with a luminometer, correlates with Caspase-3/7 activation, and luminescence was measured using a microplate reader (Molecular Devices SpectraMax 190).

ROS generation was evaluated with the ROS-Glo H_2_O_2_ assay according to the manufacturer’s protocol (Promega Biosciences San Luis Obispo, CA). The light signal produced by recombinant Luciferase enzyme is proportional to the level of H_2_O_2_ in the cells. The luminescence was measured using a microplate reader (Molecular Devices SpectraMax 190).

### Electrical Impedance Spectroscopy

The cell attachment behavior of the cells was analyzed real-time using electrical impedance spectroscopy (ECIS), a non-invasive technique that measures the impedance across gold electrodes at the bottom of tissue culture wells, using frequencies of alternating current^41,42^. Cells were plated in a 96-well ECIS array (Applied Biophysics, 96W20idf PET, Troy, NY) similar to those plated for the endpoint toxicity assays. The change in resistance at frequencies ranging from 400 to 64,000 Hz was measured over time. Low-frequency impedance can be used to monitor the solution paths around the cells, and hence the layer’s cell-to-cell barrier functions^42^. The addition of particles may complicate the impedance of the system. However, at a frequency of 4,000 Hz, the contribution of resistance through the cells was dominant and, at much higher frequencies (8,000 to 64,000 Hz), the contribution is primarily from the added particles, in this case, biomass extracts with medium^40,43,44^. Hence, a frequency of 4,000 Hz was chosen to monitor cell growth characteristics. Please refer to the Supplementary Information for more details regarding data collection and analysis.

### Statistical Analysis

Analysis of variance (ANOVA) was used to statistically compare ECIS results with the negative control (untreated cells) and positive control (medium only). A significance level of **P < 0.001 was deemed statistically acceptable.

ANOVA was also used to statistically evaluate endpoint biocompatibility testing results with a negative control. A significance level of *P < 0.05 was deemed statistically acceptable. The viability results reported in this study are normalized by the corresponding negative controls. All tissue culture data (MTT, ApoTox-Glo, and ROS-Glo H_2_O_2_) were expressed as the mean ± standard error of the mean (SEM) values of at least three independent culture experiments. For each separate cell culture experiment, 6–8 replicates were performed.

## Results

### Acute toxicity: Gas phase exposure

To assess the cellular responses to gas phase exposure, at approximately the 47^th^ hour of cell growth the viability, apoptotic cell amount, and ROS production were measured before and after exposing the cells to applewood and coal combustion emissions for one hour. The cell attachment behavior, however, was monitored in real time for more than 120 hours.

**Figure 3:**
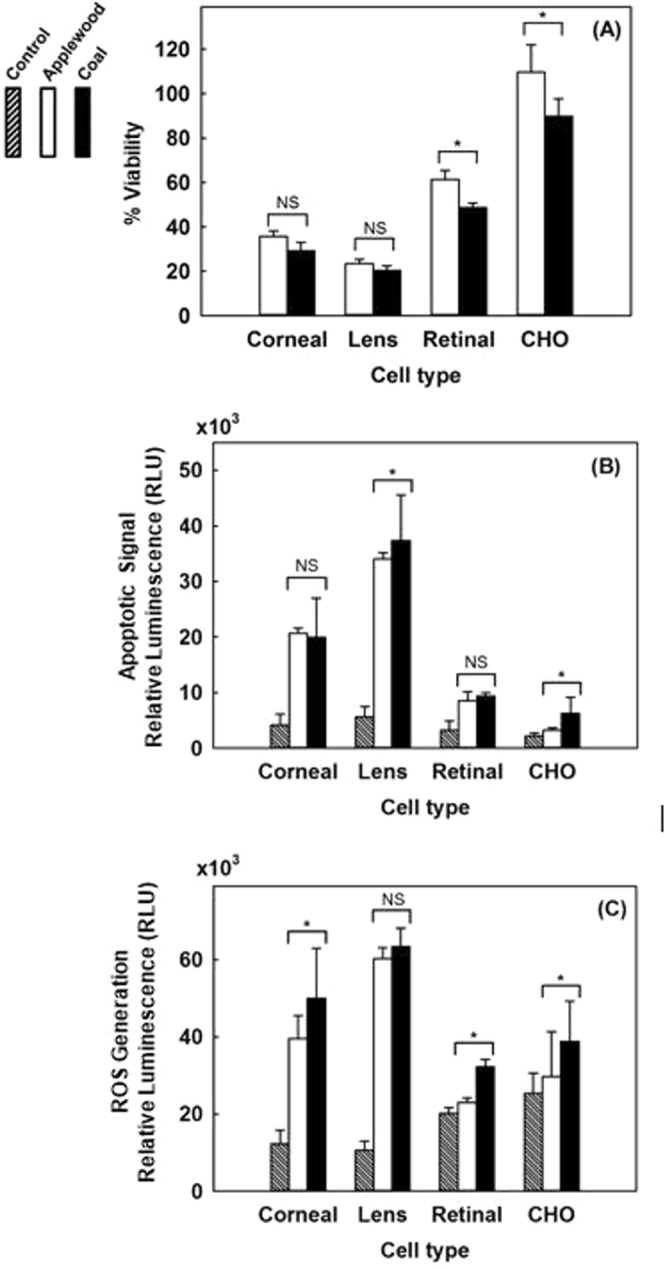
Comparative biocompatibility of applewood and biomass combustion emissions with corneal epithelial cells, lens epithelial cells, retinal pigment epithelial (RPE-19) cells, and Chinese hamster ovary (CHO) cells. (**A**) Viability results. (**B**) Amount of apoptotic cells. (**C**) ROS. Values are expressed in mean ± SEM, with each condition tested (n = 4). *P < 0.05.

Figure 3A compares the viability, amount of apoptotic cells, and the amount of ROS generation for the four cells types exposed (gas phase) to emissions from applewood and coal combustion. No significant difference was observed for the biocompatibility of corneal and lens epithelial cells exposed to the emissions from the two fuels (Fig. 3A). Both retinal and CHO cells were more biocompatible with applewood emissions than coal emissions. The viability of the CHO cells was not diminished; instead, when exposed to applewood emissions, their viability was enhanced, measuring greater than 100% of their original value. However, coal emissions adversely affected the CHO cells’ viability (89%). The apoptotic cell measurements showed statistically significant differences in the responses to applewood and coal combustion emissions (Fig. 3B). Lens epithelial cells showed the highest apoptosis signal, followed by corneal and retinal cells. The apoptotic signals recorded from retinal and CHO cells were significantly less strong than the signals from corneal and lens epithelial cells. Similar to the apoptotic signal results, corneal and lens epithelial cells produced significantly higher amounts of ROS (Fig. 3C) than retinal and CHO cells.

**Figure 4:**
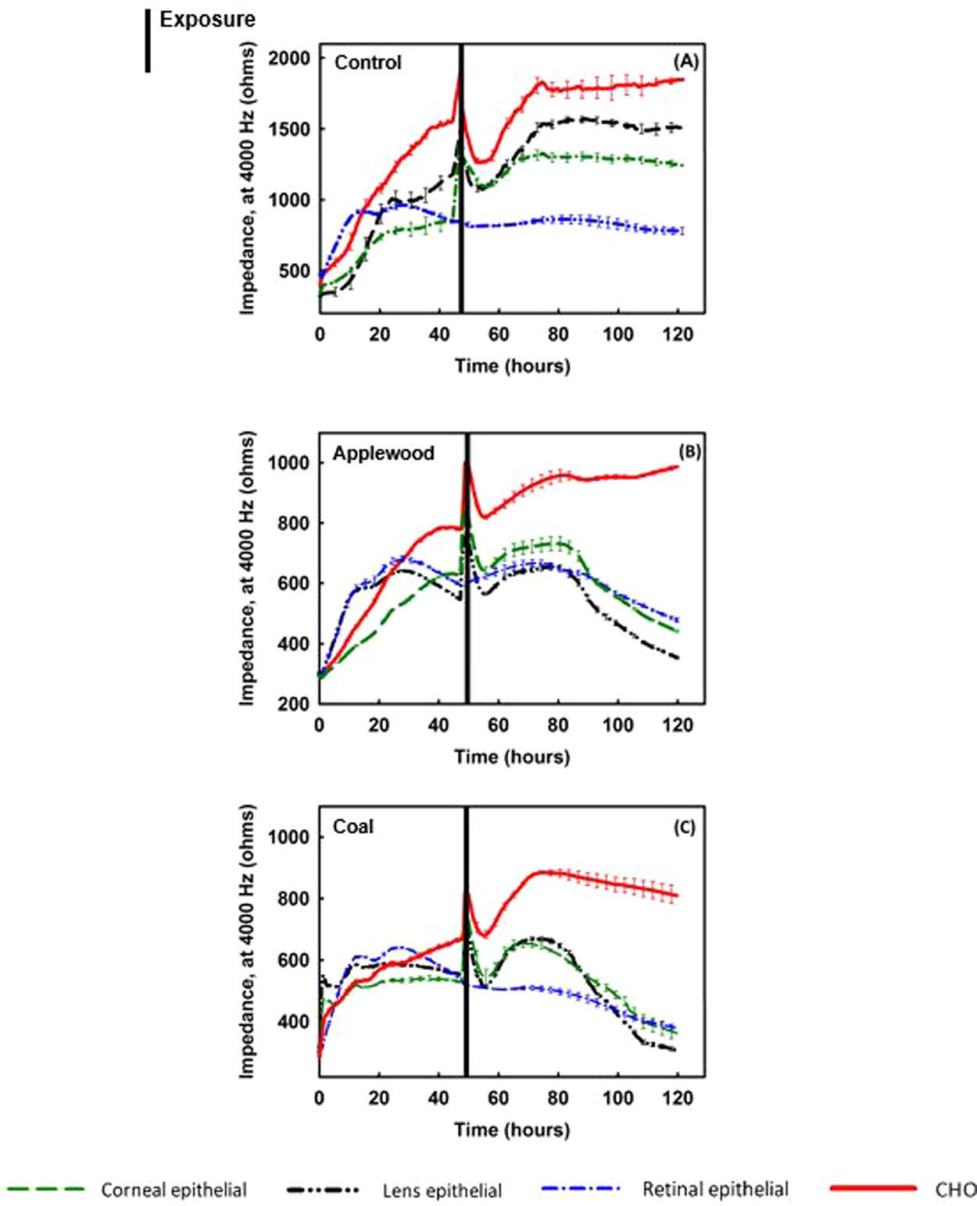
Real-time impedance measurements of corneal epithelial cells, lens epithelial cells, retinal pigment epithelial cells, and Chinese hamster ovary (CHO) cells. Cells were plated at 20,000 cells/well and exposed to stove emissions for one hour. (**A**) Control (**B**) Cells exposed to applewood combustion emissions. (**C**) Cells exposed to coal combustion emissions. Values are expressed in mean ± SEM, with each condition tested (n = 8). Black vertical lines represent the timing and the duration of exposure **P < 0.001.

ECIS allows monitoring the changes in cell morphology that are essentially evoked by alterations in the architecture of the cell’s structural components, such as the cytoskeleton and cell-cell and cell-substrate junctions^43^. Figure 4 illustrates the real-time (120 hours) impedance measurements of all cell lines tested. Figure 4A shows the results for the negative control group, the cells exposed to air only. All the cell types grew and reached confluency without showing any disruption in the impedance measures. Figure 4B demonstrates the cells’ response to applewood combustion emissions. Except for the cancer cells, all the ocular cells were adversely affected by the exposure. Similarly, all the ocular cell lines exposed to coal combustion emissions detached from the plate, resulting in a decrease in impedance. On the other hand, CHO cells maintained their confluency, although their growth rate was significantly less than that of the negative control group.

### Acute toxicity: Liquid phase exposure

Our previous study showed that emissions from coal combustion exceeded those from applewood on almost all characterization metrics, such as mass concentration, number concertation, particle size, surface area concentration, organics concentration, and carcinogenic polycyclic aromatic hydrocarbons (PAH) concentration^30^. Consistent with the above-mentioned results, the gas phase exposure study revealed that coal combustion emissions were more toxic to all cell lines tested. Therefore, for the liquid phase exposure study, only the effect of applewood combustion emission was investigated.

**Figure 5:**
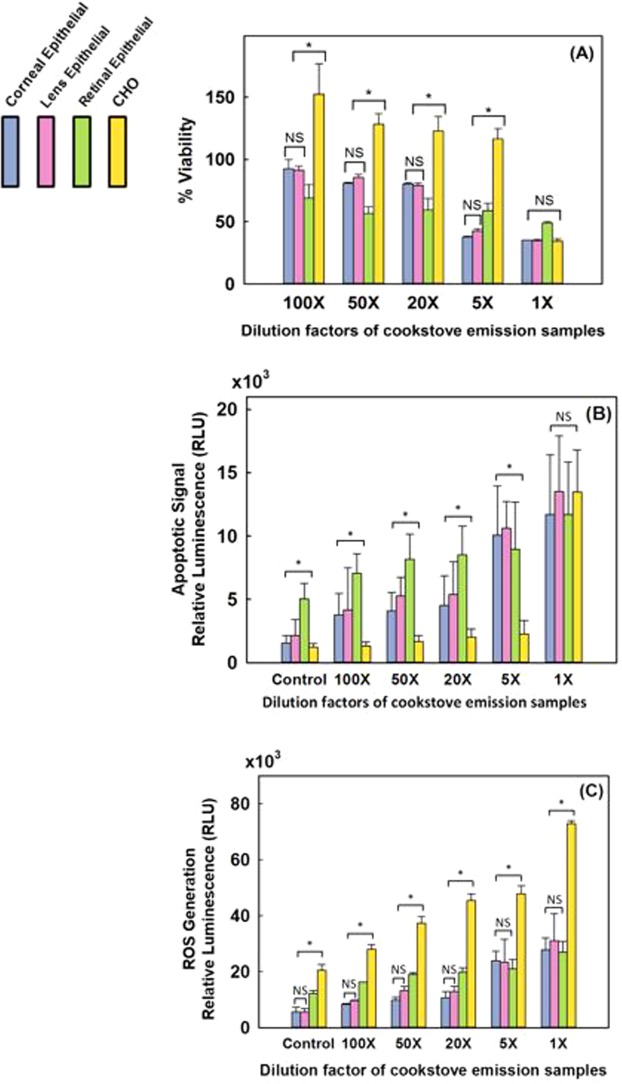
Biocompatibility measures of applewood emission extracts in contact with corneal epithelial cells, lens epithelial cells, retinal pigment epithelial (RPE) cells, and Chinese hamster ovary (CHO) cells. (**A**) Viability results. (**B**) Amount of apoptotic cells. (**C**) ROS generation. Values are expressed in mean ± SEM, with each condition tested (n = 8). *P < 0.05.

As shown in Fig. 5A, overall viability declined with increasing emission exposure levels (decreasing dilution ratio) for all healthy ocular cell types, but the viability of retinal epithelial cells demonstrated the least sensitivity to the exposure levels. Conversely, cancer cells were not affected until the exposure concentration was increased to a dilution of 1:1. Interestingly, except for the 1:1 dilution, the cancer cells showed more than 100% viability, i.e., their growth was promoted. Unlike the overall viability results, retinal cells sent the strongest signals of impeding apoptosis until the exposure concentration was increased to a dilution of 1:20 (Fig. 5B). On the other hand, the amount of ROS generation in healthy ocular cells increased with increasing exposure concentration (until a dilution of 1:20), and similar to the apoptosis signaling results, retinal cells generated relatively more ROS than corneal and lens epithelial cells (Fig. 5C).

In addition to the experimental endpoint toxicity evaluations of viability, apoptosis, and ROS generation, electrical impedance spectroscopy (ECIS), a sensing technique for monitoring cell motion and morphological changes real-time, was also used^45,46^. Upon plating, cells start to attach to and spread out over the gold electrodes. As a result, the impedance increases because the insulating cell membranes form tight junctions that block the electrical current’s flow. Conversely, when cells are stressed and dying, cell-to-cell junctions are disrupted. Moreover, changes in cell morphology, such as contractile rounding of membranes and detachment of cells from the gold electrode, result in higher electrical current passage, which leads to a decrease in impedance over time^47^.

**Figure 6:**
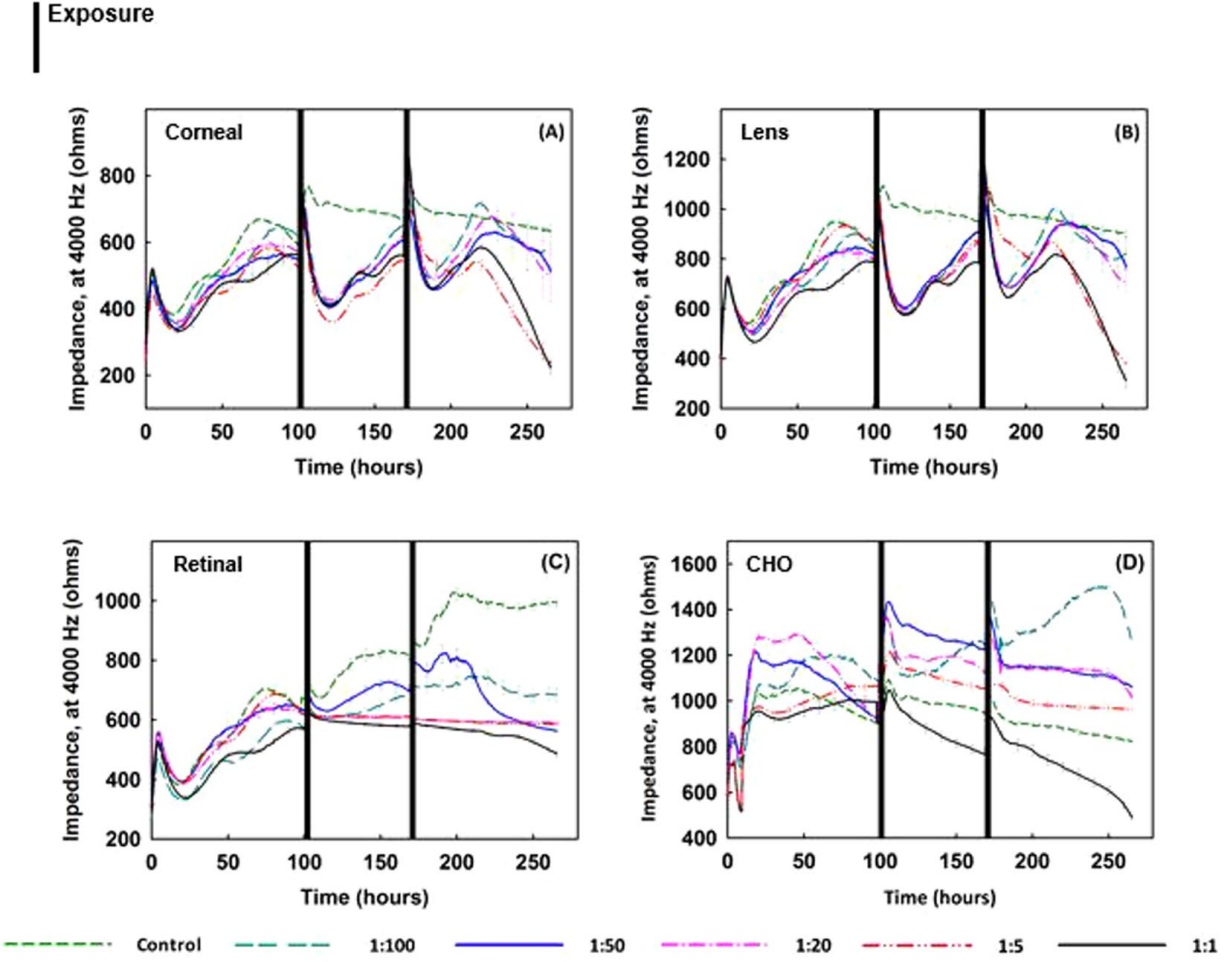
Impedance measurements of (**A**) corneal, (**B**) lens, (**C**) retinal epithelial, and (**D**) CHO cells plated at 20,000 cells/well, exposed to applewood emission extracts with different dilution rates. Note that after the cells become confluent, the medium was changed only once throughout the rest of the lengthy exposure period. As compared to the control cells, the healthy cells, which were exposed to the extracts, could not recover, except at the highest dilution rate (1:100). Overall, the applewood emission extract did show a toxic effect on ocular cells; however, the same concentrations that were toxic to healthy cells accelerated the growth of CHO cells. Values are expressed in mean ± SEM, with each condition tested (n = 8). **P < 0.001.

Figure 6 presents electrical impedance measurements for all cell types tested. Cells were allowed to reach confluency (~97 hr) and then exposed to emission extracts twice, once at the 97^th^ hour, and again at 168^th^ hour, latter had recovered and again reached confluency. The real-time response behavior for corneal epithelial (Fig. 6A) and lens epithelial cells (Fig. 6B) was similar. For both cell types, after the initial response to exposure, a sharp decline was recorded in impedance during the first 20 hours post-exposure for all exposure levels tested. The impedance level recovered following both the first and second exposures; however, eventually, it dropped gradually. After the second exposure, the decline was irreversible. The impedance levels of both cell types (Fig. 6A,B) continuously declined irreversibly below the initial impedance level, based on the data collected up to the 265^th^ hour, the end of the experiment. It was also observed that the rate of the final decline in impedance was higher for higher exposure levels. With higher exposure concentrations, it is likely that the cells reacted more drastically. In what follows, it is evident that there is a complex pattern of growth followed by retardation and resumption as a result of exposure to biomass smoke. Most notably, however, the retinal cells were markedly less affected (Fig. 6C). The growth rate of the retinal cells was significantly affected; however, the cells still maintained a confluent layer by the end of the experiment. Finally, for the cancer cells, except at the highest exposure concentration, the impedance levels were higher than for the control cells (Fig. 6D). Interestingly, when the cells were exposed to the extracts with the lowest dilution ratio, the impedance level of the cells was recorded to be 53% more than the impedance level of the unexposed cells (the negative control) by the end of the experiment (265^th^ hour).

## Discussion

To better understand the effect of indoor air pollutants on the eye, it is important to estimate the particulate deposition efficiency on the ocular surface. There have been several studies on estimating the deposition velocity of the particulate matter, which is one of the main factors on the degree of deposition efficiency^48^. The other factors that play a role in the deposited amount of particulate matter on the ocular surface are the orientation of the flow, humidity, and the degree of turbulence^48,49^. The time-dependent rate of particle deposition depends on the airborne particle concentration, the particle deposition velocity, and the exposed ocular surface area^50^. On the other hand, the physiological defense mechanism, the tear film, plays a critical role in removing the deposited particles^51^. Therefore, the accumulated amount of particles on the ocular surface is the net result of deposition from the air and removal by the tear film^50^. As a result, the estimation of the net amount of particulate matter deposited on the ocular surface is rather complex. The purpose of the present study was to assess the extent of ocular cells’ as well as cancer cells’ response to gas and liquid phase biomass and coal combustion exposures. A follow-up study, currently being planned, will investigate the *in-vivo* response to biomass combustion exposure while considering the deposition efficiency of particulate matter for the unprotected eye in the indoor environment.

Apoptosis, programmed cell death, is a complex phenomenon^52^, to date, various pathways were reported that are involved in its initiation^53–55^. A biphasic effect of ROS has been demonstrated to promote cell proliferation at lower doses^55^; however, once a certain threshold is exceeded^20^, it causes organelle damage and initiate cell death^54,55^.

In the light of our findings, biomass smoke leads to the generation of ROS, and once a certain threshold is exceeded; the cells were no longer able to scavenge the excessive ROS. This excessive amount of ROS retarded the growth of healthy ocular cells and eventually killed them. Among the non-cancerous healthy cell lines tested, retinal cells showed the greatest resistance to bio-induced ROS generation. In fact, the CHO cancer cells stood out in their resistance: For both gas and liquid phase exposures, biomass smoke promoted their growth. The specifics are discussed below.

### Acute toxicity: Gas phase exposure

*In-vitro* studies with biomass and coal smoke can potentially generate toxic products from reactions between the smoke components and cell media^56,57^. Nevertheless, for both practical and ethical reasons, evaluating emissions on cells is a logical precursor to *in-vivo* tests. In conformance with strict animal protocols, animals, unlike cells, cannot be exposed to biomass and coal emissions for extended periods^56^. Furthermore, the specific way a cell reacts to its environment varies with the set of receptor proteins on its cell membrane and according to their primary role in the body^58^. Cell types have evolved to perform different roles, and thus their responses to the same stimuli can be dissimilar^59^. *In-vitro* models can advantageously provide results for longer exposure periods and multiple types of cells.

In Fig. 3A, we compare the viability, amount of apoptotic cells, and the amount of ROS generation for the four cells types exposed (gas phase) to emissions from applewood and coal combustion. Corneal and lens epithelial cells exposed to the emissions from the two fuels showed similar viability results, and the type of fuel did not show a statistically significant difference (Fig. 3A). On the other hand, both retinal and CHO cells were more tolerant of applewood emissions than coal emissions. Surprisingly, when exposed to applewood emissions, CHO cells actually grew, their viability was recorded as 109% (Fig. 3A). Unlike applewood emissions, coal emissions did adversely affect the CHO cells’ viability (89%). The apoptotic cell measurements showed statistically significant differences in response to applewood and coal combustion emissions for lens epithelial and CHO cells only (Fig. 3B). The amount of apoptotic cells was highest among the lens epithelial cells, followed by corneal and retinal cells. Simply put, cells succumb to environmental stress differently^59,60^. The concentration of applewood and coal smoke that led to apoptosis in corneal and lens epithelial cells did not result in the same level of apoptotic response in retinal and CHO cells. Our results also showed that retinal pigment epithelial (RPE) cells can cope well with oxidative stress (Fig. 3C). This result may be attributed to their unique ROS handling capacity, an evolutionary mechanism for tolerating light exposure^61^. Similar to the apoptotic signal results, corneal and lens epithelial cells produced significantly higher amounts of ROS (Fig. 3C). On the other hand, retinal and CHO cells showed relatively less ROS generation as a result of gas phase exposure to biomass and coal emissions. As expected, since the ROS generation was higher in corneal and lens epithelial cells, apoptosis signal initiation was higher in those cells and evidently was reflected in the eventual viability results (Fig. 3). Overall, based on viability, apoptosis, and ROS generation results, coal appears to show more adverse effects than applewood combustion emissions on all cell lines tested.

Smoke from solid-fuel combustion is a complex mixture of hundreds of organic and inorganic chemical species in both gaseous and particulate phases, making it very difficult to associate health effects with a single component. More details on smoke constituents and their associated health effects can be found in^62^, where the authors reviewed relevant studies from 1980 to 2016, with a focus on China. Detailed physical and chemical characterizations of the PM emissions from the two fuels tested in this study have been published in our previous studies^30,31^, and therefore are not included here. Emissions from coal combustion exceeded those from applewood on almost all characterization metrics, such as mass concentration, number concentration, particle size, surface area concentration, organics concentration, and polycyclic aromatic hydrocarbons (PAH) concentration^30^. Therefore, it is not possible to comment on the role of any single metric in the trends observed in Fig. 3. Systematic and controlled laboratory studies are required to associate different specific emission components and metrics with their health effects.

Figure 4A shows real-time cell attachment impedance measurements for all four cell types when removed from incubation conditions and exposed to filtered air (negative control). For all cell types except retinal cells, impedance levels decline sharply just after exposure to filtered air, but eventually recover to their original value. The variations in the initial drop in the impedance might be due to the cells’ varied abilities to compensate for and adapt to the changes in the extracellular environment^63,64^. It is clear that retinal cells are least affected by changes in, for example, the amount of carbon dioxide and the temperature, and therefore their impedance level is least altered (Fig. 4A).

Figures 4B,C show the responses of all cell types to applewood and coal emissions, respectively. The initial post-exposure responses of cells were similar to those for the control, i.e., a decline in impedance, followed by recovery. Again, this decline may be due to the cells’ varied abilities to adapt to the extracellular environment. Unlike the control experiments, the impedance dropped for retinal, lens, and corneal epithelial cells, starting at the 73^rd^, 78^th^, and 81^st^ hours, respectively. However, the impedance level of CHO cells continued to rise until the end of the experiment (~120 hours) (Fig. 4C). This increase is a result of cell proliferation, triggered by the increased amount of intracellular ROS (Fig. 5C), as reported previously^20,21,65^. It can, therefore, be concluded that for CHO cells, the increased ROS caused more cell proliferation^66^.

### Acute toxicity: Liquid phase exposure

The retinal cells withstood increased oxidative stress better than the corneal and lens epithelial cells (Fig. 5A,C). ROS production in cells is a double-edged sword^67^. ROS are created mainly as host defenses against infectious agents; however, when a threshold is exceeded, increased ROS can lead to apoptosis^65^. We measured both the apoptotic cell amount as well as ROS generation and found a direct correlation for healthy ocular cells (Fig. 5B,C). When the amount of ROS production exceeded the cells’ capacity to repair oxidative damage, the cells initiated apoptosis, as was also reported in previous studies^68,69^. On the other hand, cancer cells adapted to oxidative stress. Their viability results were significantly higher than for the healthy ocular cells (Fig. 5A). Comparing the apoptosis onset values measured in RLU (Fig. 5B) with the ROS values in Fig. 5C, it can be seen that cancer cells tolerate 1.7–2.7 times greater concentration of ROS than healthy ocular cells. This finding is consistent with previous studies that reported significantly higher ROS generation in cancer cells than in healthy cells^65,69^. Notably, our apoptosis measurement technique is based on Caspase 3/7 activity measurement, which is detectable only during apoptosis, not during necrosis^70^. Apoptotic cell death is a programmed cell death as a result of increased oxidative stress (ROS generation); therefore, there is a strong correlation between ROS generation and the apoptotic cell amount (Fig. 5).

Unlike the corneal and lens cells (Fig. 6A,B), the real-time impedance results for retinal epithelial cells did not show a sharp decline following the exposure events (Fig. 6C). The first exposure did not affect the impedance; however, after the second exposure, the impedance decreased slightly. The cells did not crash by the end of the experiment because, as explained previously, retinal cells can cope with oxidative stress better than other ocular cell types. Interestingly, the impedance measurements of the cancer cells revealed an unexpected behavior. The CHO cells which were exposed to cookstove emissions extracts grew more than the control cells, displaying the same unexpected behavior found with gas phase exposure, which suggests that exposure to biomass exhaust can promote tumor growth.

As a topic for a future *in-vitro* study, it would be interesting to investigate the effect of particle size on the response of both healthy ocular cells and cancerous cells. Deposition velocity, one of the most significant factors on the deposited amount of particulate matter on the ocular surface, has been shown to vary within an order of magnitude for 0.1 µm particles, and by much more for larger particles^50^. An *in-vitro* study can be designed to investigate the effect of particle size by filtering a certain range of particles; i.e., sub 100 nm, sub 1 µm, sub 10 µm, and sub 100 µm, and exposing them to both ocular and cancer cells.

Although we do not fully understand exactly how inhaling stove emissions causes secondary injury to the eye and other organs^16,29^, extended exposure to smoke efficiently transfers environmental pollutants into the bloodstream^71,72^. Due to their high metabolic activity, tumor cells need more oxygen and nutrients than normal cells^73^. It has been shown that tumor cells can grow their own blood vessels^74^, and as a result, tumors receive more blood than normal cells^75,76^. Once environmental pollutants enter the bloodstream, their constituents inevitably reach tumor cells. Our *in-vitro* study demonstrates that once tumor cells are in direct contact with biomass emissions, their growth is promoted. Based on our findings, it is reasonable to hypothesize that daily exposure to biomass smoke from stoves poses a risk for cancer patients.

## Conclusions

Our *in-vitro* study found a significant disturbance in healthy ocular cells’ attachment, indicative of death, as a result of both liquid and gas phase biomass smoke exposure. On the other hand, the growth of cancer cells was significantly promoted. The ROS amount that led to ocular cell death did not destroy the cancer cells, but instead accelerated their growth. Our *in-vitro* study connects cellular responses with epidemiological disease findings, clearly underscoring the troubling implication that people exposed daily to biomass-fueled stove smoke are at risk, especially considering that our findings were for a limited exposure time. People who are regularly exposed to biomass combustion smoke are more prone to developing eye diseases, either from direct exposure or secondary exposure by inhalation. More importantly, everyday activities like cooking and heating using stoves with biomass fuels may accelerate the growth of tumors. These findings emphasize that we should pay added attention to the environment of the person living with cancer, not only to their genetic predisposition.

## Supplementary information

Investigating the Effects of Stove Emissions on Ocular and Cancer Cells

## Data Availability

The authors declare that all the relevant data supporting the findings of this study are available in the article or from the corresponding author upon request.
