# Supplementary material for: Investigating the Effects of Stove Emissions on Ocular and Cancer Cells
Source: Sci Rep. 2019 Feb 12;9:1870. doi: 10.1038/s41598-019-38803-4 (PMC6372759; doi:10.1038/s41598-019-38803-4)
Supplement: Supplementary file 1 — Investigating the Effects of Stove Emissions on Ocular and Cancer Cells [file 41598_2019_38803_MOESM1_ESM.docx]

Supplementary Information

Investigating the Effects of Stove Emissions on Ocular and Cancer Cells

Bedia Begüm Karakoçak^1^, Sameer Patel^1^, Nathan Ravi^1,2,3^, Pratim Biswas^1^*

^1^Department of Energy, Environmental, and Chemical Engineering, Washington University in St. Louis, St. Louis, MO 63130, USA.

^2^Department of Ophthalmology and Visual Sciences, Washington University in St. Louis, St. Louis, MO 63110, USA.

^3^Veterans Affairs St. Louis Hospital, St. Louis, MO 63106, USA.

*Correspondence to Pratim Biswas.

**Email:** pbiswas@wustl.edu **Tel: +**1-314-935-5548 **Fax: +**1-314-935-5464

**Table S1.** Relative humidity measurements throughout the biomass/coal exposure experiments.

| Time (hours) | % RH | |
| --- | --- | --- |
|  | **Gas Phase** | **Liquid Phase** |
| 0 | 83.1 | 85.1 |
| 10 | 85.2 | 85.3 |
| 20 | 84.3 | 84.9 |
| 30 | 84.2 | 83.5 |
| 40 | 80.0 | 87.2 |
| 47 | 67.0 | 85.1 |
| 50 | 83.1 | 84.7 |
| 60 | 85.2 | 86.0 |
| 70 | 84.3 | 83.9 |
| 80 | 83.3 | 84.5 |
| 90 | 82.9 | 85.2 |
| 101 | 83.1 | 65.2 |
| 110 | 84.0 | 85.2 |
| 120 | 84.5 | 86.5 |
| 130 |  | 83.7 |
| 140 |  | 83.8 |
| 150 |  | 84.1 |
| 167 |  | 76.3 |
| 170 |  | 86.0 |
| 180 |  | 84.8 |
| 190 |  | 85.0 |
| 200 |  | 85.5 |
| 210 |  | 86.1 |
| 220 |  | 85.2 |
| 230 |  | 84.2 |
| 240 |  | 83.2 |
| 250 |  | 84.7 |
| 260 |  | 85.0 |

***Cell Morphology Photographs for Confluency Cross-check***

**
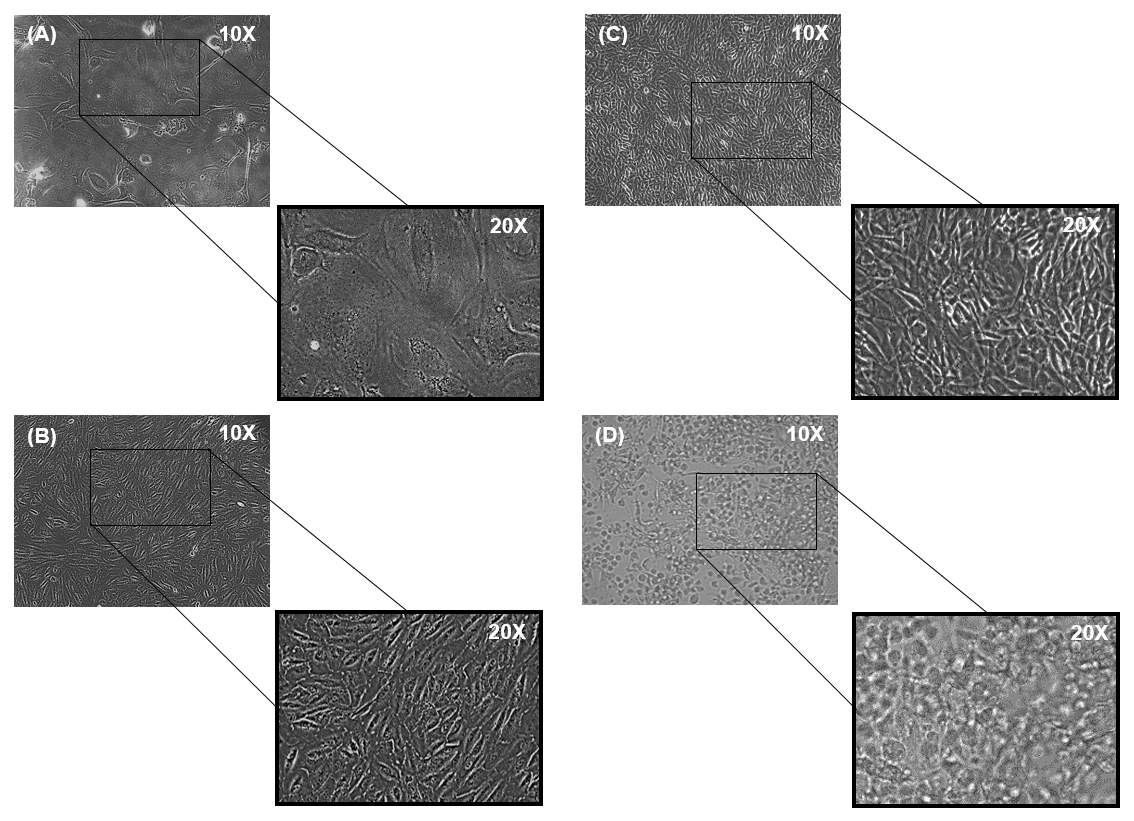
**

**Figure S1.** The images of confluent cell monolayers were obtained using an Olympus CKX41 inverted microscope with ×10 and ×20 magnification. The visualization window is 60 × 60 μm.

***Cell Morphology Photographs before and after Exposure to Applewood and Coal Smoke***

**
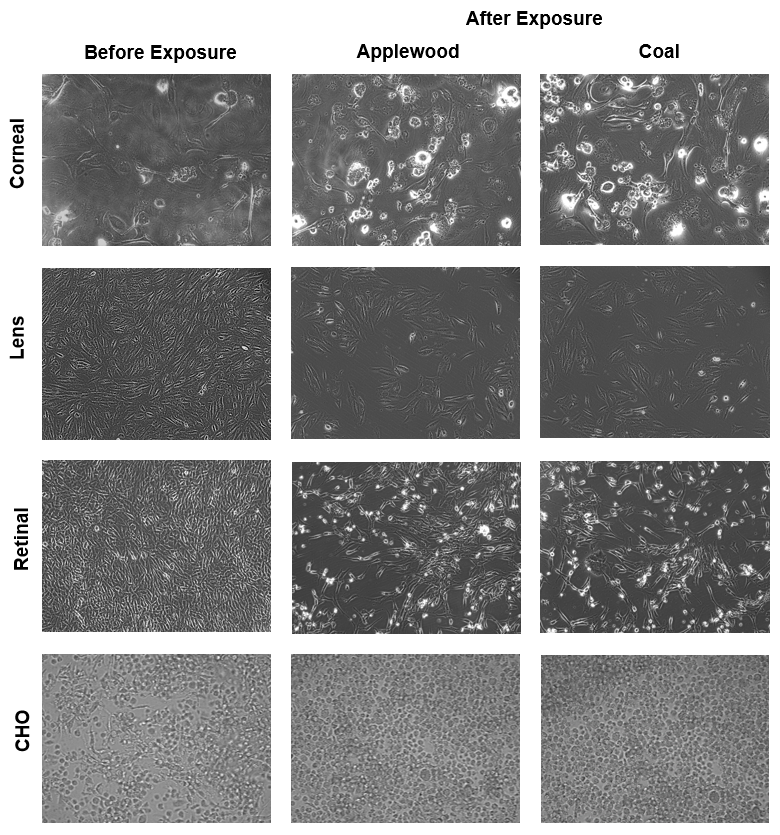
**

**Figure S2.** Cell morphology images of all tested cell lines before and after exposure to applewood and coal smoke were obtained using an Olympus CKX41 inverted microscope with ×10 magnification. The visualization window was 60 × 60 μm. Corneal cells became rounder after exposure to smoke. Whereas lens and retinal cells became detached and were washed away. CHO cells, on the other hand, multiplied after exposure, crowding the plate more densely.

***Electrical Impedance Spectroscopy (ECIS) Data Collection and Analysis***

*Data Collection*

The cell attachment behavior of the cells was analyzed in real-time using electrical impedance spectroscopy (ECIS), a non-invasive technique that measures the impedance across gold electrodes at the bottom of tissue culture wells (Figure S3A), using alternating current frequencies ^1,2^. (Figure S3). Cells were plated in a 96-well ECIS array (Applied Biophysics, [96W20idf PET](http://www.biophysics.com/cultureware.php#link3), Troy, NY) (Fig. S4A). In an instrument called and ECIS Station, a 96-well ECIS array is placed in the tissue culture incubator space (37^o^C, 5% CO_2_) (Fig. S4B).The ECIS instrument can simultaneously measure both the resistance and capacitance of the ECIS electrodes over AC frequencies from 400 Hz to 64,000 Hz. Impedance data was collected every ~18 minutes for the entire frequency range (Fig. S4C).

**96-well plate**

**Gold electrodes embedded underneath each well**

**(A)**


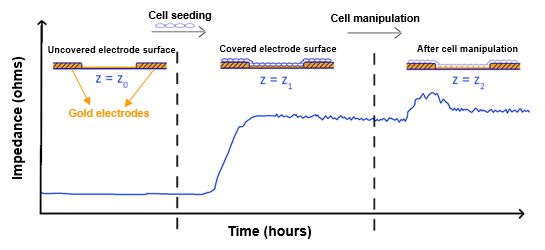

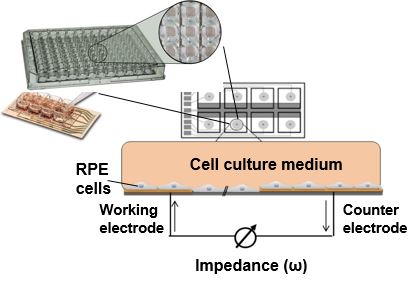


**Confluency reached**

**(B)**

**Figure S3.** ECIS working principle. (A) Plate configuration. (B) The representative sketch is showing data collection phases (the illustrations are adapted with permission from Applied Biophysics: <http://www.biophysics.com/>).


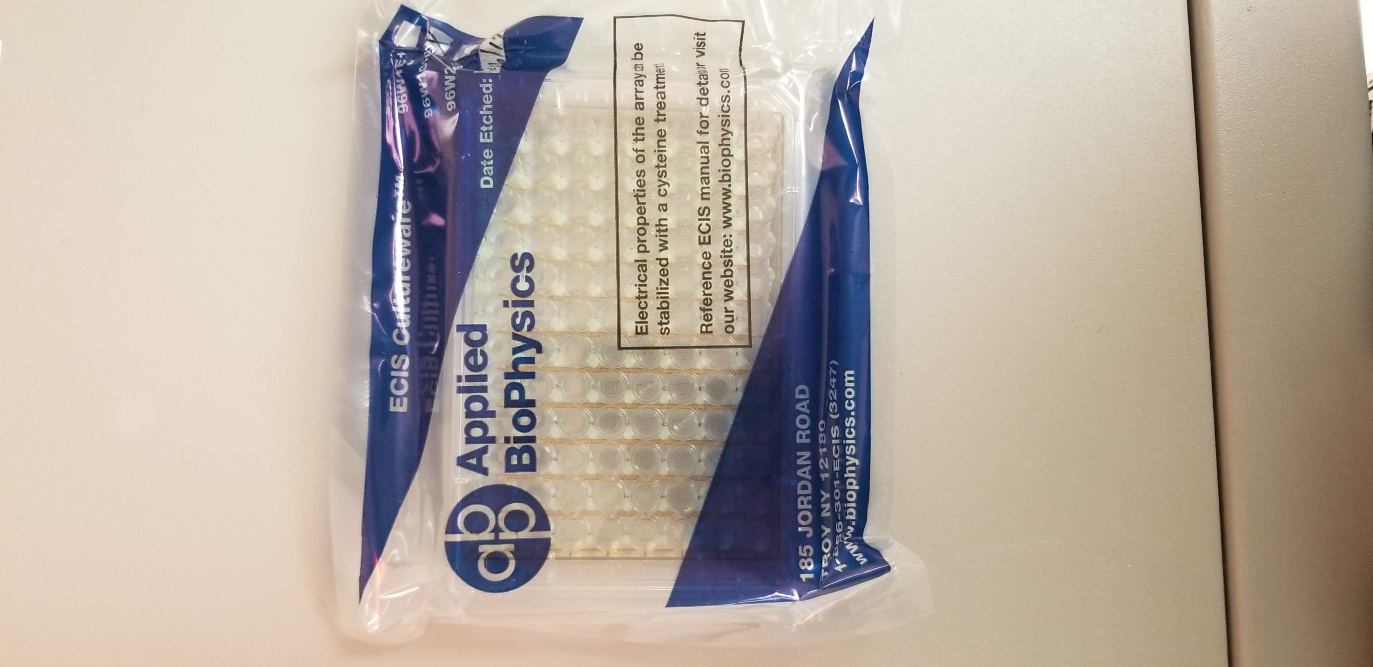

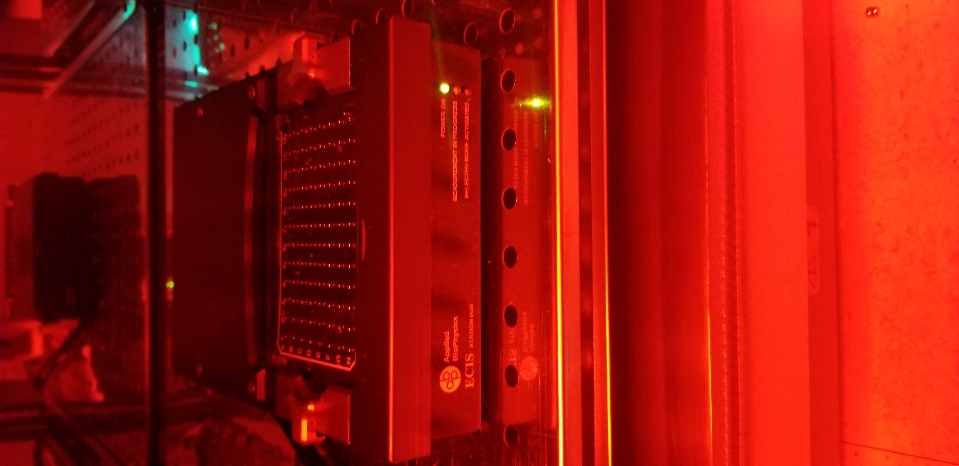


**(B)**

**(A)**

**(C)**


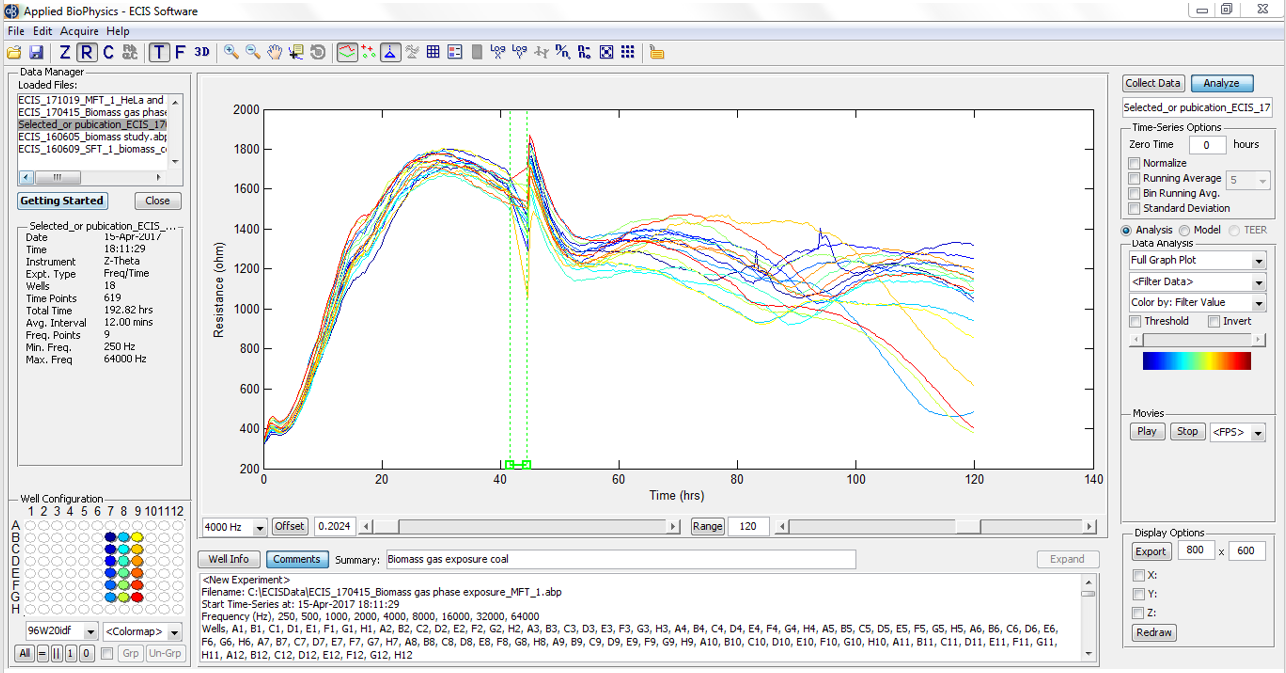


**Figure S4.** (A) A typical ECIS 96-well plate (Applied Biophysics, [96W20idf PET](http://www.biophysics.com/cultureware.php#link3), Troy, NY). (B) ECIS 96-well plate holder inside an incubator. (C) A snapshot figure from ECIS Applied Biophysics Software (V1.2.163) for collected raw data. Each column represents a different cell type: for example, column 9 indicated by a red arrow in the lower left screen panel, represents lens epithelial cells exposed to applewood smoke.

*Analysis*

Upon completion of the experiment, the results were normalized by the cell-free wells (positive controls). Next, the contents of the wells holding the same type of cells were extracted and analyzed separately (Figure S4C). For each condition, there were 6-8 replicates. The raw data was retrieved, normalized by the impedance readings with the wells with media only (positive control), and the standard error was then calculated. Analysis of variance (ANOVA) was used to statistically compare ECIS results with the negative control (untreated cells) and positive control (medium only). A significance level of ***P* < 0.001 was deemed statistically acceptable.

**References**

1. Arndt, S., Seebach, J., Psathaki, K., Galla, H. J. & Wegener, J. Bioelectrical impedance assay to monitor changes in cell shape during apoptosis. *Biosen. Bioelectron*. **19**, 583-594 (2004).

2. Wegener, J., Keese, C. R. & Giaever, I. Electric cell-substrate impedance sensing (ECIS) as a noninvasive means to monitor the kinetics of cell spreading to artificial surfaces. *Exp. Cell Res.* **259**, 158-166 (2000).
